# Medical Student Research Journals: The International Journal of Medical Students (IJMS) Legacy

**DOI:** 10.5195/ijms.2022.1467

**Published:** 2022-04-05

**Authors:** Kiera Liblik, Patricio Garcia-Espinosa, Ahmed Nahian, Surobhi Chatterjee, Mihnea-Alexandru Găman, Ciara Egan, Juan C. Puyana, Francisco J. Bonilla-Escobar

**Affiliations:** 1 BSc. Faculty of Medicine, Queen’s University, Kingston, ON, Canada. Associate Editor, IJMS; 2 Social Service Medical Doctor. School of Medicine, Universidad Autónoma de Nuevo León, Monterrey, México and Mexican Institute of Social Security. Palliative Care Unit. High Specialty Medical Unit #25, Monterrey, México. Student Editor, IJMS; 3 BS/DO Medical Student. California Baptist University-Lake Erie College of Osteopathic Medicine, Riverside, CA. United States. Student Editor, IJMS; 4 MBBS, Intern Doctor. Department of Medicine, King George’s Medical University, Lucknow, Uttar Pradesh, India. Student Editor, IJMS; 5 MD, PhD student. Faculty of Medicine, “Carol Davila” University of Medicine and Pharmacy, 050474 Bucharest, Romania & Department of Hematology, Center of Hematology and Bone Marrow Transplantation, Fundeni Clinical Institute, 022328 Bucharest, Romania. Scientific Editor, IJMS; 6 Medical Student. Humanitas University, Humanitas Research Hospital, Milan, Italy. Deputy Editor, IJMS; 7 MD, FRCSC, FACS, FACCP. School of Medicine, Department of Surgery, Professor of Surgery, Critical Care Medicine, and Clinical Translational Science, Director for Global Health-Surgery, University of Pittsburgh, Pittsburgh, PA, United States. Editorial Board Member, IJMS; 8 MD, MSc, PhD(c). Researcher, Department of Ophthalmology; Institute for Clinical Research Education (ICRE), University of Pittsburgh, Pittsburgh, PA, United States. CEO, Fundación Somos Ciencia al Servicio de la Comunidad, Fundación SCISCO/Science to Serve the Community Foundation, SCISCO Foundation, Cali, Colombia. Grupo de investigación en Visión y Salud Ocular, VISOC, Universidad del Valle, Cali, Colombia. Editor in Chief, IJMS

As the longest-standing, non-interrupted, International Journal for Medical Students with a high impact, visibility, and an international inclusive editorial board, the objective of the International Journal of Medical Students (IJMS) is to be the primary diffusion platform for early-career scientists in medicine, using evidence-based standards in the process of scientific publication.^1^ It is crucial that medical students are valued and credited for their work which, in turn, can lead to tremendous impact on the quality of research output generated and education of the next generation of the global medical-scientific community. This objective has been met since 2013 with the first issue of the IJMS, starting from a unique idea reached during a discussion at an international congress of medical students in 2009.^1,2^

Since, the history of the IJMS has been anything but meager. It has achieved a significant milestone sought by emerging medical journals worldwide; the indexing of a publication in PubMed Central (PMC).^2^ A group of researchers, including medical students, were funded by the National Institute of Health (NIH) of the United States and chose IJMS for their high-quality article.^3^ This demonstrates that, indeed, a journal focusing on medical students and created and edited by themselves is not at odds with quality, despite traditional misconceptions.^4^

Times are changing. The process of scientific production has traditionally ‘punished’ medical students, in the words of Corral-Reyes, I.^5^ The process that the medical student must carry out is even greater than those that some renowned authors must take, even if they are practically the same. The publication process is complicated not because of the lack of quality but because of the stigma around their scientific production. Although, as the same author emphasizes, there is a lack of valuation of their own work and lack of expertise when it comes to perceiving how, when, and where to publish; the absence of a publication culture.^5^

Therefore, it is necessary to encourage medical student journals to value student growth and commitment to research, giving rise to space for visualization and training. This culture began in Latin America in 1961 with the Cuban magazine “16 de Abril” (April 16).^5^ This tradition of more than 60 years is precisely what has opened and seeded the path for journals like the IJMS to flourish in a difficult and sometimes arid environment for medical student research.

What started as an international project from Latin-America has become a Journal whose team represents 34 different nationalities across all the continents, made up of researchers, mentors, and experts in various fields of medicine. The IJMS focuses on the growth and expansion of the scientific medical-student community. Thus, creating a space not only for sharing science and innovation, but a voice behind the experiences, failures, and hardships inclusive and representative of the diverse, dynamic, medical student global community. Moreover, it is important to highlight that all Student and Associate Editors of the IJMS, as well as its Executive Committee, are graduates of the peer-review training courses offered by Web of Science Academy.

The scientific literature includes a wide range of medical student journals, pursuing different objectives, goals, and strategies to achieve their respective aims. An overview of these journals is shown in Table 1 (excluding those that do not publish original research).

Although the success of these journals has been noteworthy and has been framed by decades of continuous publication, others, sadly, have not been able to prevail to the challenge of publishing scientific articles by medical students. Particularly, these journals have the tasks of reviewing, editing, and publishing; which sometimes must be accompanied by correction, education, and teaching of the next generation of medical scientists. This is not a small task and has led to the demise of these journals. Among them we can find the Medical Student Journal of Australia, Trinity Student Medical Journal, Asian Student Medical Journal Genesis, MJM, International Journal of Students’ Research, Dares Salam Medical Students’ Journal, Scottish Universities Medical Journal, Acta Cientifica Estudiantil, Esculapio, SCEMUSS, SCientifica, among others.^27,28^

Though the IJMS is published in English for ease of integration into mainstream literature, the IJMS has a tremendous advantage in that our diversity of authors encompasses 39 different countries in the past year alone.^26^ Accordingly, we are newly integrating a summary for non-scientific audiences of each article in the language where the research was carry out. This serves to streamline the translation of scientific knowledge, allowing easy access for knowledge users in the context in which the research was conducted.

The present issue, composed of 16 articles, showcases work by authors from a wide variety of countries, including India, Mexico, the Philippines, the United Kingdom, Kenya, Ireland, and different parts of the United States. Authors include students, early career researchers, and mentors with impressive academic qualifications. To believe that because a journal is formative, orienting, and attractive to medical students, that it is less impactful is to proverbially judge a book by its cover. Some of the greatest revolutions in medicine and beyond were led by trainees and the IJMS aims to be one such in providing academic representation to students.

In this issue, we are publishing 10 original research papers: 7 original articles, 1 short communication, 1 review, and 1 case report. In addition, we are publishing 6 experiences from medical students worldwide that could be of help when facing the realities of medical education. The contents of this work is summarized, as follows.

In addition, we are publishing six experiences from medical students worldwide to aid in understanding the realities of medical education during a time of global unrest. First, an editorial about the war on Ukraine and how this is impacting medical education in the country. This is the first time that the IJMS has published an editorial on political conflict. Though, as discussed in the previous IJMS volume, medical students are global citizens and affected by global situations, such as wars and climate change.^29–31^ It is critical that these issues be discussed and addressed. In our editorial we make a call for violent conflict to be halted and to use discussion and collaboration in the context of political discourse.^32^

Due to the high degree of reported distress experienced by medical trainees, it is critical that reliable metrics are developed to elucidate key stressors in this population. Thus, medical student Montano et al. sought to determine the reliability of the Medical Student Stressor Questionnaire. They determined that the reliability of the questionnaire is excellent and that stressors varied by sex.^33^ In terms of measuring perceived competency, recent medical school graduate Canton et al. assessed the efficacy of a surgical scrubbing, gowning, and gloving checklist for trainees. Their checklist had high inter-rater reliability and internal consistency.^34^ Another interesting study on training, a cross-sectional study conducted with the fifth-year medical student Nidhi Thomas, showed that of students who chose an elective rotation, the minority pursued a specialty in that discipline.^35^ Notably, Huang et al. found that one of the barriers to matching to specialties is socioeconomic inequality impacting interviews due to connection and audio problems.^36^ It is similarly important that the perspectives of medical educators be integrated in the evaluation of education. Educators have had to adapt to online teaching during the COVID-19 pandemic, with a lack of adequate warning or training. Final-year medical student Andrew Thomas collaborated with a team of investigators to determine educators’ attitudes to online learning. They reported a need for better infrastructure to support interactive learning in an online format. Interestingly, almost half of the participants supported continued online learning.^37^

Beyond online learning, telecommunication is one of the most utilized medical tools during the COVID-19 pandemic. Park et al. describe the role of telerehabilitation as a safe, accessible, efficient, and comfortable alternative to in-person interventions for people with spinal cord injuries.^38^ The pandemic has also influenced bedside care. Accordingly, Farley et al. present an epidemiological profile of a pediatric hospital before and during the COVID-19 pandemic. They describe a significant decrease in the number of patients admitted for respiratory conditions and speculate the reasons for this stark change.^39^ Another study focused on pediatric medicine was conducted by Murerwa et al., critically reviewing the literature on prenatal and postnatal mercury exposure due to skin lightening agents with inorganic mercury. The authors advocate that prevention is the only way to reduce mercury poisoning and toxicity.^40^

Patient advocacy is an important role of the medical professional, including gender diverse patients. Bonasia K, et al. highlighted differences in access to healthcare for transgender and gender-diverse patients. Their article sought to determine knowledge and perception on the subject by medical students and institutions. They conclude that clinical skills were less valued when dealing with non-binary patients as compared to a cis-gender patients.^41^ Another point where improvement must be made in medical education is in teaching on commonly missed and misdiagnosed diseases. Urs et al. present a case on Dyke-Davidoff-Masson syndrome, a commonly missed and serious cause of refractory epilepsy which requires an understanding of pertinent imaging and clinical reasoning.^42^ Interestingly, although ischemic heart disease is the leading global cause of death there is a lack of literature discussing the predictors of early versus late readmission to hospital following discharge for an ischemic event. Third-year medical student George Cholack et al. conducted a retrospective study of patients hospitalized for acute coronary syndrome and found that female patients were more likely to have late rehospitalization as well as non-white individuals, and those who initially required intensive care unit admission. This information can be used to inform follow-up after ischemic heart events, aiming to reduce morbidity and mortality.^43^

Finally, important perspectives of medical trainees are highlighted. Patricio Garcia-Espinosa shares his experience as the first cohort of undergraduates allowed to rotate in the palliative care ward in Mexico. His impactful description of the role of palliative medicine, the need for undergraduates to learn and rotate in this specialty, and its inclusion in the undergraduate curriculum is worth reflecting upon.^44^ Similarly, Waisberg shares an experience of an “eye opening” mission trip to an underserved community in Montemorelos, Mexico that provided him new contacts, mentors, networking possibilities, and novel cultural experiences in different nations are all important insights into a specialty.^45^ On the other side of the world, Rocha et al. describes an experience of post-graduate interns helping their community by participating in COVID-19 vaccination drives in the Philippines, gaining practical knowledge and hands-on experience.^46^ Patel et al. also describe inaccessible and inequitable care, but for dermatological disease in underrepresented and underserved communities forming the basis of the Student Dermatological Clinic for the Underserved and a collaborative service-learning model in Pittsburgh.^47^ Another student initiative, Mulwalkar describes the journey of creating a student-oriented research and innovation council, ASPIRE, in India.^48^

We hope that you enjoy reading this issue as we did in making it a reality. This is a tremendous effort of more than 70 team members volunteering to make the vision of showcasing medical students research a reality.

## Figures and Tables

**Table 1.** Medical Student Research Journals Around the World.

JOURNAL	COUNTRY	FOUNDATION	UNINTERRUPTED ACTIVE YEARS	ORGANIZATION	READERS PER YEAR	H-INDEX**	H5-INDEX**
**16 de Abril** ^22^	Cuba	1961	1962-2022	Universidad de Ciencias Médicas de la Habana	15802 users, 60000 readers	3	4
**AMSJ** ^7^	Australia	2009	2010-2021	UNSW	17,000; more tdan 5000 email subscribers, 2000 copies for all Australian med schools	N/D	N/D
**AMSRJ** ^15^	USA	2013	2014-2020	LSU Shreveport	N/D	N/D	N/D
**ANACEM** ^22^	Chile	2007	2007-2021	ANACEM	N/D	4	6
**Científica Ciencia Médica** ^22^	Bolivia	1994	1997-2021	Universidad Mayor San Simón	23505 users, 61519 readers (2021)	5	6
**CIMEL** ^22^	Peru	1995	1995-2022	FELSOCEM	N/D	3	6
**CRMJ** ^16^	USA	2018	2018-2022	Cooper Medical School of Rowan University	4000 per year	N/D	N/D
**DMJ** ^20^	Canada	1936	2003-2022	Dalhousie University	3440 Total	N/D	N/D
**FMSRJ** ^9^ *	USA	2015	2016-2020	Herbert Wertheim College of Medicine.	N/D	N/D	N/D
**IJMS** ^12^	USA	2012	2013-2022	University of Pittsburgh	71,000 users (2021), 263,383 pageviews	9	16
**Médica MD** ^22^	Mexico	2009	2009-2022	Universidad de Guadalajara	N/D	5	7
**Médicas UIS** ^22^	Colombia	1987	1987-2022	Universidad Industrial de Santander	54374 users, 72,000 readers (2021)	5	10
**NZMSJ** ^18^	New Zealand	2003	2004-2021	University of Otado/ University of Auckland	N/D	N/D	N/D
**SJHR-AFRICA** ^23^	Uganda	2020	2020-2022	HENU, Healtd Nest Uganda, Student’s Health Research, Africa Limited	N/D	4	6
**UBCMJ** ^21^	Canada	1962	2009-2022	University of British Columbia	N/D	N/D	N/D
**UTMJ** ^10^	Canada	1923	1923-2022	University of Toronto	N/D	5	6

JOURNAL	MOST READ ARTICLE (reads, Last name)	MOST CITED (citations, Last name)	INDEXING	LANGUAGE	PERIODICITY	PUBLICATION TYPE
**16 de Abril** ^22^	1923, Arman-Pereda, D.^23^	11, Guilarte. ^24^	Google Scholar, IMBIOMED, LATINDEX, BIBLAT, MEDIGRAPHIC	English and Spanish	Triannual	Electronic
**AMSJ** ^7^	N/D	19, Nguyen M.^8^	Google Scholar	English	Biannual	Electronic and Print.
**AMSRJ** ^15^	N/D	N/D	N/A	English	Annual	Electronic and Print
**ANACEM** ^22^	N/D	N/D	Latindex, Imbiomed, Index Copernicus, EBSCO, LILACS, Google Scholar, Academic Journals Database	English and Spanish	Biannual	Electronic and Print
**Científica Ciencia Médica** ^22^	N/D	N/D	SciElo Bolivia, Redalyc, DOAJ, Latindex, Redib, EBSCO, ROAD, Lilacs, Dialnet, MIAR, Crossref, Imbiomed, Google Scholar	Spanish	Biannual	Electronic and Print
**CIMEL** ^22^	N/D	N/D	DOAJ, REDIB, Imbiomed, OAJI, DRJI, Google Scholar, Latindex, Lilacs, Europub, Google Scholar	English and Spanish	Biannual	Electronic and Print
**CRMJ** ^16^	1228, Ellis, S.^17^	N/D	DOAJ, Google Scholar	English	Annual	Electronic and Print
**DMJ** ^20^	N/D	N/D	N/D	English	Biannual	Electronic
**FMSRJ** ^9^ *	N/D	N/D	N/D	English	Annual	Electronic.
**IJMS** ^12^	6810, Rondilla, et al. ^13^	54, Bawazeer NA^14^	BASE; DOAJ; EZB; Google Scholar, HINARI, IMBIOMED, OCLC, J GATE	English	Quarterly	Electronic
**Médica MD** ^22^	N/D	N/D	Imbiomed, EBSCO, AMERBAC, LATINDEX, REDIB, MEDIGRAPHIC, ACADEMIC ONE FILE	English and Spanish	Triannual	Electronic and Print
**Médicas UIS** ^22^	N/D	N/D	SciElo Colombia, LILACS, REDIB, Dialnet, EBSCO, Hinari, Periodica, Imbiomed, Publindex	English and Spanish	Quarterly	Electronic and Print
**NZMSJ** ^18^	N/D	18. Al-Busaidi I.^19^	Google Scholar	English	Biannual	Electronic and Print
**SJHR-AFRICA** ^23^	N/D	N/D	DOAJ, Google Scholar, Science Gate, OUCI	English	Quarterly	Electronic
**UBCMJ** ^21^	N/D	N/D	N/D	English	Biannual	Electronic
**UTMJ** ^10^	N/D	32, Cape J.^11^	Scopus, Google Scholar	English	Triannual	Electronic and Print

Considering only Journals with Original Articles and Medical Students as authors.

AMSJ: Australian Medical Student Journal, AMSRJ: American Medical Student Research Journal, ANACEM: Asociación Nacional Científica de Estudiantes de Medicina, CIMEL: Ciencia e Investigación Médica Estudiantil Latinoamericana, CRMJ: Cooper Rowan Medical Journal, DMJ: Dalhousie Medical Journal, FMSRJ: Florida Medical Student Research Journal, FMSRP: Florida Medical Student Research Publications, IJMS: International Journal of Medical Students, NZMSJ: New Zealand Medical Student Journal, SJHR-Africa: Students’ Journal of Health Research Africa, UBCMJ: University of British Columbia Medical Journal, UTMJ: University of Toronto Medical Journal.

* The FMSRJ was published from 2015 to 2020 and then changed to Cureus journal/publications under a channel named FMSRP.

** H-INDEX and H5-INDEX obtained from Google Scholar Statistics: available at: https://scholar.google.com.cu/citations?view_op=metrics_intro&hl=en. For journals that were not available in Google Scholar, the statistics provided by the journal’s own web page were consulted, or searched through the international bibliography.

Those data not found are marked with the legend N/D: No Data.
